# Adaptive bill morphology for enhanced tool manipulation in New Caledonian crows

**DOI:** 10.1038/srep22776

**Published:** 2016-03-09

**Authors:** Hiroshi Matsui, Gavin R. Hunt, Katja Oberhofer, Naomichi Ogihara, Kevin J. McGowan, Kumar Mithraratne, Takeshi Yamasaki, Russell D. Gray, Ei-Ichi Izawa

**Affiliations:** 1 Department of Psychology, Keio University, Tokyo, Japan; 2 School of Psychology, University of Auckland, Auckland, New Zealand; 3 Auckland Bioengineering Institute, University of Auckland, Auckland, New Zealand; 4 Department of Mechanical Engineering, Keio University, Yokohama, Japan; 5 Cornell Lab of Ornithology, Ithaca, New York, USA; 6 Yamashina Institute for Ornithology, Abiko, Japan; 7 Max Planck Institute for the Science of Human History, Jena, Germany

## Abstract

Early increased sophistication of human tools is thought to be underpinned by adaptive morphology for efficient tool manipulation. Such adaptive specialisation is unknown in nonhuman primates but may have evolved in the New Caledonian crow, which has sophisticated tool manufacture. The straightness of its bill, for example, may be adaptive for enhanced visually-directed use of tools. Here, we examine in detail the shape and internal structure of the New Caledonian crow’s bill using Principal Components Analysis and Computed Tomography within a comparative framework. We found that the bill has a combination of interrelated shape and structural features unique within *Corvus*, and possibly birds generally. The upper mandible is relatively deep and short with a straight cutting edge, and the lower mandible is strengthened and upturned. These novel combined attributes would be functional for (i) counteracting the unique loading patterns acting on the bill when manipulating tools, (ii) a strong precision grip to hold tools securely, and (iii) enhanced visually-guided tool use. Our findings indicate that the New Caledonian crow’s innovative bill has been adapted for tool manipulation to at least some degree. Early increased sophistication of tools may require the co-evolution of morphology that provides improved manipulatory skills.

Human tool behaviour is underpinned by adaptations associated with enhanced manipulation of tools and tool material. For example, human shoulder and hand characteristics that allowed high-speed throwing and enhanced hand manipulation, respectively, probably evolved in *Homo erectus* 2–1.4 Ma^1^,^2^. Such morphological specialisation may have played a crucial role in enabling the subsequent increased sophistication of Acheulean stone tool technology^2^. There is little positive support for this hypothesis from nonhuman primates as their tool making is generally rather simple^3^ and there is no evidence that they have morphology adapted to tool behaviour. However, the complex tool skills of the New Caledonian crow (NCC, *Corvus moneduloides*) may be underpinned by a suite of morphological, behavioural and cognitive adaptations^4^.

The NCC is one of the few species with a tool-using lifestyle where tool making and use is practised year round throughout the species’ range^5^. The skill with which NCCs manipulate tools is impressive and shown by their technique to ‘fish’ large longhorn beetle grubs out of their burrows in dead wood^6^,^7^. To do this, some individual NCCs first use the tool tip to irritate a grub by touching sensitive areas around its large mandibles. When the grub reacts aggressively by moving its mandibles, the bird then holds the tip at the mandibles ready to extract the grub when it bites the tool. Furthermore, the level of complexity of the NCC’s tool manufacture in the wild is unrivalled among nonhumans^8^. On mainland Grande Terre birds craft hooked implements out of live twigs^8^,^9^,^10^ and remove several standardized hooked tool designs from the barbed edges of *Pandanus* spp. leaves using their bills^8^,^11^,^12^. No other nonhuman species incorporates hook technology into tool designs for foraging in the wild^3^,^8^.

The species-level tool lifestyle^5^, the genetic disposition for basic tool skills^13^,^14^, the dexterity of tool making and use and the complexity of tool designs together suggest that the NCC’s tool behaviour has evolved over a very long period of time. A potential morphological adaptation associated with the NCC’s tool behaviour is the shape of its bill, which is noticeably straight for a *Corvus* species^15^,^16^. In a qualitative investigation, we visually confirmed from images that all *Corvus* species except the NCC had decurved bills (Supplementary Table S1, Fig. S1). That is, the bills curve downwards at their distal ends. It has been suggested that a straight bill enables enhanced visually-guided manipulation of the working end of a tool^15^. The NCC appears to have separated from its closest *Corvus* relative around 5 Ma^17^, providing sufficient time for bill morphogenesis to occur^18^. However, there has been no in-depth study of the shape and internal structure of its bill to examine if it has been adapted for tool manipulation.

Here, we investigated if the NCC’s bill morphology has been adapted for tool behaviour. Our study had two complimentary parts. The first part set out to (i) determine the important shape characteristics that make the NCC’s bill unique among *Corvus* species, and (ii) characterise specific shape features that might be adaptive for tool manipulation. To do this, we first compared the bill shapes of 10 *Corvus* species and a woodpecker species using landmark data in a Principal Components Analysis (PCA) and carried out an analysis of bill curvature. We included a woodpecker because the NCC has a woodpecker-like niche in New Caledonian forests where it extracts invertebrate prey from live and dead trees^7^,^19^. In the second part of the study we compared the internal structure and cross-sectional shape of the NCC’s bill and two other *Corvus* species with different foraging behaviour (the rook *C. frugilegus* and the large-billed crow *C. macrorhynchos*) based on Computed Tomography images.

## Results

**Figure 1:**
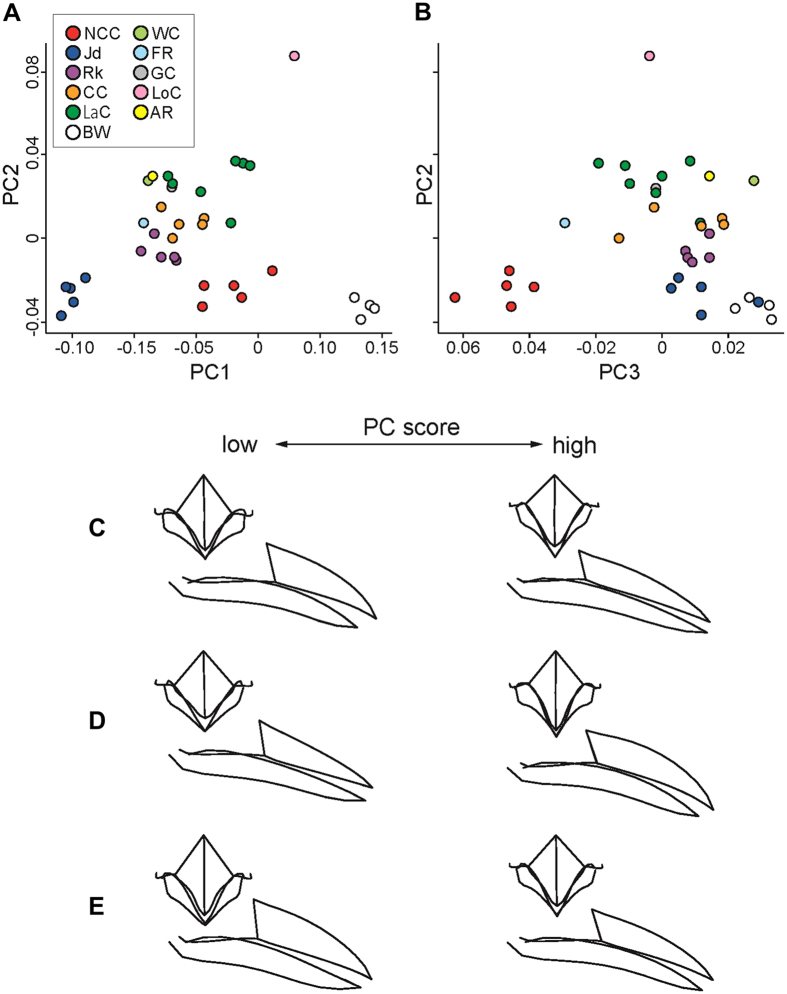
Results of the Principal Components Analysis of bill shape. (**A**,**B**) Scatter plots of Principal Components Analysis outputs. (**C–E**) Principal Component-based wireframe models of the bill. Inter-species differences in bill shape are shown in PC1-PC2 (**A**) and PC2−PC3 (**B**) space. The wireframe models for PC1 (**C**), PC2 (**D**) and PC3 (**E**) indicate how each of the three significant Principal Components contributes to the overall bill shape when the values of all other Principal Components are held constant. NCC, New Caledonian crow; Jd, Daurian jackdaw; Rk, rook; CC, carrion crow; LaC, large-billed crow; WC, white-necked crow; FR, fan-tailed raven, GC, gray crow; LoC, long-billed crow; AR, Australian raven; BW, black woodpecker.

Based on the 11 primary landmarks and 81 semi-landmarks located on each bill, there was obvious variation in bill morphology between the 11 species in the PCA (Fig. 1). PC1, PC2, and PC3 explained 59.18%, 10.78%, and 7.87% of total variance, respectively. PC1 separated the two outlier species, the Daurian jackdaw (*C. dauuricus*) and the black woodpecker (*Dryocopus martius*), from the other nine (*Corvus*) species (Fig. 1A,C). Jackdaws, genetically distant from other *Corvus* species^17^,^20^, have shorter, stubby bills with the upper mandible longer than the lower mandible. In contrast, the black woodpecker has a long, slender bill with the lower mandible longer than the upper mandible^21^. PC2 separated the black woodpecker, the Daurian jackdaw and the NCC from the other species on the degree of bill curvature and length of the upper mandible (Fig. 1A,D). PC3 importantly separated the NCC from all of the other species, based on the depth of the upper mandible and a straighter, upwardly inclined lower mandible (Fig. 1B,E). For all three principal components, the PC scores for the six species with *n* > 1 were significantly different (species: NCC, Daurian jackdaw, rook, carrion crow *C. corone*, large-billed crow and black woodpecker; PC1: *F*_5,25_ = 80.50, *p* < 0.001; PC2: *F*_5,25_ = 58.94, *p* < 0.001; PC3: *F*_5, 25_ = 34.86, *p* < 0.001). The five *Corvus* species in these ANOVA tests represent Clades I, IV and VI-VIII (Supplementary Fig. S2). While the NCC is the *Corvus* species with a bill shape closest to that of the black woodpecker (Fig. 1A) because of its straightness, its bill also has features very different from the bill of a specialist woodpecker, such as a deeper profile and an upturned lower mandible (Fig. 1B).

**Figure 2:**
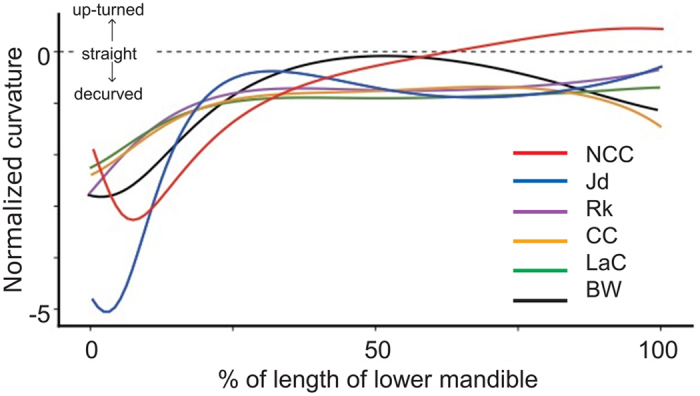
Mean normalized curvature profiles of the superior edge of the lower mandible in five *Corvus* and one woodpecker species. A curvature value smaller than 0 indicates decurvature, equal to 0 straightness, and greater than 0 an upturned profile. The tip of the mandible is the 100% mark on the x-axis. The five *Corvus* species represent Clades I, IV and VI-VIII (see Supplementary Fig. S2). The NCC was the only species with an upturned lower mandible. *n* = 6 for the large-billed crow and *n* = 5 for the other species. The key to the species abbreviations is in the legend to Fig. 1.

The tomium of the caudal region of the NCC’s lower mandible is decurved (from 0 to around 40% of its length), similarly to that of other *Corvus* species and the black woodpecker (Fig. 2). Then it becomes straighter in the mid region, as in the black woodpecker, before being unique in inclining upwards to the bill tip.

**Figure 3:**
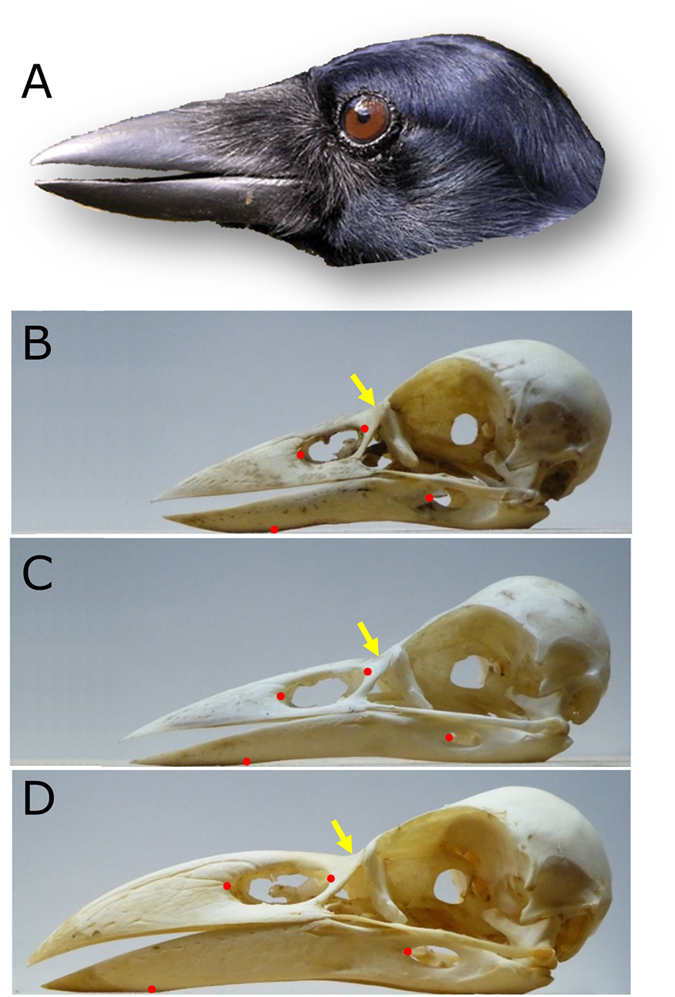
Bill shape of the three *Corvus* species used in the analysis of bill structure. (**A**) The head of a NCC. (**B**) The skull of NCC 2. (**C**) The skull of Rook 2. (**D**) The skull of LaC 1. Arrows indicate the approximate position of the nasal-frontal hinge where the upper mandible joins the cranium. Dots on the upper mandible show the edges of the nasal openings. On the lower mandible they indicate the rostral edge of the *Fenestra rostralis* (at right) and the gonydeal angle where the rami join at the posterior end of the keel. Length differences between the three skulls indicate actual differences (the distance from the nasal-frontal hinge to the tip of the upper mandible for NCC 2 = 4.3 cm long, for Rook 2 = 4.9 cm, and for LaC 1 = 6.0 cm).

Visual comparison of the NCC’s bill with those of the rook and the large-billed crow reveals distinct differences between the three species (Fig. 3). The NCC’s bill is more stout and wedge-shaped with only a slightly curved culmen (i.e., the ridgeline of the upper mandible). The rook’s bill is relatively slender, with a moderately curved culmen. The bill of the large-billed crow is relatively large with a strongly curved culmen. The NCC’s bill has characteristics that the rook and the large-billed crow lack. In particular, the tomium of the upper mandible is relatively straight but the tomium on the lower mandible has an upwards incline along much of the keel section. The base of the upper mandible caudal to the nasal openings is deeper, which reduces the curvature of the culmen. The keel is noticeably more upturned compared to the rook and the large-billed crow and takes up a greater percentage of the length of the bill. Visual comparison of Computed Tomography cross-sectional images confirm that the NCC’s bill has a relatively deeper upper mandible, a deeper profile along the middle of the keel on the lower mandible and wider rami also on the lower mandible (see selected images from each of the 3 species in Supplementary Fig. S3). The depth of the NCC’s upper mandible at the caudal edge of the nasal openings is very similar to that of the large-billed crow even though the NCC has a much shorter bill. These features provide a stout bill with a relatively square profile that should better withstand forces in all directions.

**Figure 4:**
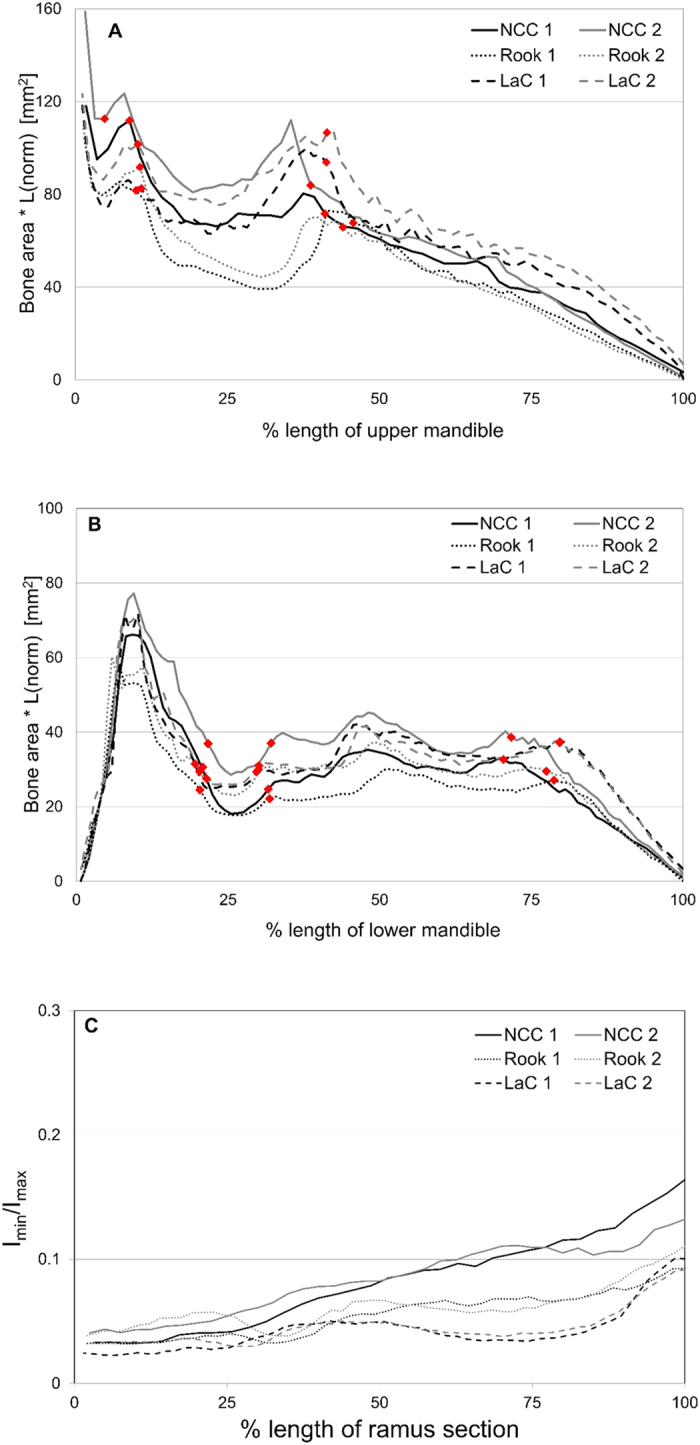
Comparison of shape and internal structure of bills between NCCs (NCC 1, NCC 2), rooks (Rook 1, Rook 2) and large-billed crows (LaC 1, LaC 2). (**A**,**B**) Normalised total bone area along the upper and lower mandibles. (**C**) I_min_/I_max_ values along the right ramus of the lower mandible between the rostral edge of the *Fenestra rostralis* and the gonydeal angle. Diamonds on (**A**) indicate edges of the nasal openings and on (**B**) indicate the edges of the *Fenestra rostralis* (at left) and the gonydeal angle (at right).

The relative amount of bone in the bills of all three species is similar in both the upper and lower mandibles (Fig. 4A,B). The two rooks have less bone between the caudal and rostral edges of the nasal openings than the NCCs and the large-billed crows, with NCC 2 having the most bone in this region. NCC 2 also seems to have more bone along much of the lower mandible. The position of the nares relative to the length of the upper bill is very similar for all three species (Fig. 4A), but the positions of the gonydeal angles confirm that the keel of the NCC is relatively longer compared to that of the rook and the large-billed crow (Fig. 4B). I_min_/I_max_ values confirm that the rami of the NCCs become more circular towards the keel compared to the rami of the rooks and the large-billed crows (Fig. 4C). Thus the shapes of the mandibular rami in the NCC should be better able to withstand horizontal as well as vertical bending forces.

The ratio of in-lever to out-lever distance was significantly larger in the NCC (mean = 0.19, s.d. = 0.015, *n* = 5) compared to that in the rook (mean = 0.17, s.d. = 0.004, *n* = 5) and large-billed crow (mean = 0.17, s.d. = 0.004, *n* = 5) (*F*_2, 12_ = 7.74, *p* < 0.01). The larger ratio in the NCC indicates that its bill is relatively shorter than the bills of the other two species and thus capable of a relatively stronger bite.

## Discussion

We found that the NCC’s bill has a unique combination of morphological characteristics within the genus *Corvus*, predominately associated with shape rather than increased bone area. The shape features are (i) a relatively deep, short, stout bill, (ii) particularly straight tomia, especially on the upper mandible, (iii) an upward sloping tomium at the front of the lower mandible, (iv) wider mandibular rami with increased resistance to horizontal bending forces, and (v) a relatively long, deep and upturned keel of the lower mandible. Although our morphological analysis did not include all *Corvus* species or detailed biomechanical analysis, our initial study is the first to confirm the unique morphology of the NCC’s bill. In the following we argue that the NCC’s bill morphology is specialised for tool use and at least some of the above features evolved for enhanced tool manipulation after the species began using tools.

Bill morphology in birds is a combination of both phylogeny and adaptation for species-specific task requirements^22^. The uniqueness of the NCC’s bill within *Corvus*, along with the NCC’s more recent speciation within its genus^17^, indicates that its novel features have evolved through species-specific adaptations. The main species-specific, interrelated factors affecting bill shape are: (i) the feeding method, (ii) the forces acting on the bill, (iii) the necessary size of the bill, and (iv) the weight of the bill^22^. Given that the NCC’s bill appears to be relatively short for a *Corvus* species, we only consider the first two factors. Island passerines generally have longer bills than their close continental relatives^23^,^24^. This pattern is associated with wider ecological niches, possibly as a response to increased competition for food; longer bills are advantageous when probing in tree holes and under bark for prey^24^. Birds in tropical regions might also be expected to have larger bills to help dissipate excess heat^25^. However, the NCC’s relatively deep, short bill suggests that its shape is unlikely to be a specialisation for probing or a tropical climate. The relatively short out-lever distance compared to in-lever distance on the lower mandible also means slower tip closing velocity, making it more difficult to catch fast moving prey^26^. The straight tomia would be inefficient for cracking hard objects like seeds given that birds with this behaviour have very short, strongly decurved bills to provide greater crushing force^22^,^27^.

Two, not necessarily mutually exclusive, behaviours could have been important in evolving the NCC’s novel bill morphology. The first is clearly tool use, which provided a much greater extended reach for probing compared to evolving a longer bill. The second behaviour is using the bill directly to extract difficult-to-access prey from wood and forest vegetation. NCCs have a foraging niche similar to that of a woodpecker’s based on extraction of mostly invertebrate prey from trees^7^,^19^. They also sometimes drive their bills forcefully into decayed wood when excavating a hole to get a longhorn beetle grub^7^. This action is done with the bill slightly open to grasp and remove pieces of wood to get access to prey (GRH pers. obs.). In contrast, the more specialised woodpecker species, which dig into hard wood and have the straightest bills in the Picidae, use a slender, closed bill to chisel into wood^22^, as represented in PC1−PC2 coordinates in the landmark analysis (Fig. 1). Less specialised woodpeckers (e.g. the rufous piculet, *Sasia abnormis*) that do not excavate hard wood but can forage in softer, decaying wood like NCCs do have more decurved bills than the more specialised woodpeckers^28^. Birds other than woodpeckers that specialise in excavating wood to capture prey appear to have either decurved or recurved bills. For example, three South American species (Peruvian and Bolivian recurvebills, *Syndactyla* spp. and the recurve-billed bushbird, *Clytoctantes alixii*) that appear to open up hollow wood with their bills to obtain insect prey have bills where both the lower and upper tomia curve upwards^29^,^30^. The New Zealand kaka (*Nestor meridionalis*) specialises in extracting longhorn beetle grubs out of dead wood by using its strongly decurved parrot’s beak to dig into wood^31^.

**Figure 5:**
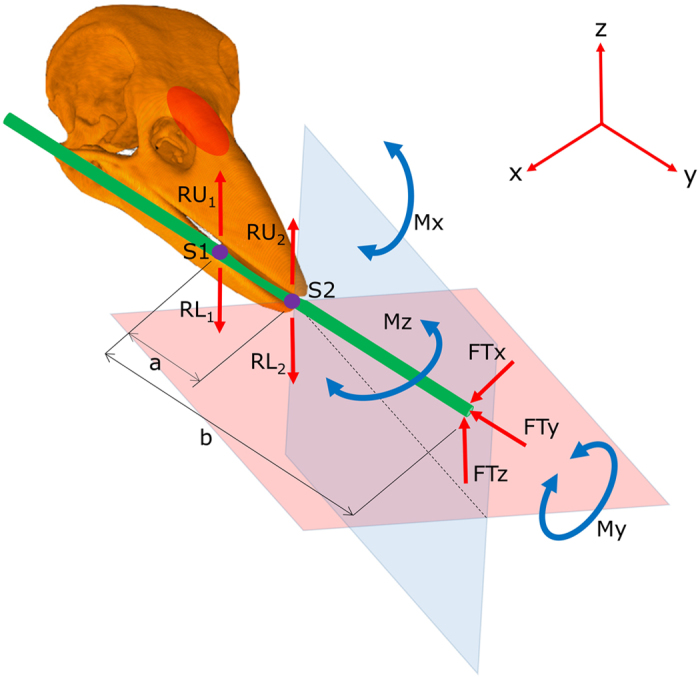
Simplified diagram showing the main forces and moments acting on the NCC’s bill associated with using a tool for probing. The 3D reconstruction of a NCC’s skull is shown with a tool held angle-ways in the bill, mostly on the right side of the front of the bill and at the bill tip. The straight bill enables the tip of a straight tool to be positioned on the mid-frontal plane of the bill (pink plane) and as close as possible to the mid-sagittal plane of the bill (blue plane). The dashed black line indicates the assumed ideal position of the tool tip for its visually-directed manipulation. Probing with the tool tip creates a unique set of forces and moments that act on the bill in three dimensions. Since the moment arms of the tool tip force FT are functions of distances *a* and *b*, as *b* increases the moments acting on the bill become large quickly. The tool must be held securely by increased reaction forces acting on the bill at S1 (RU_1_ and RL_1_) and S2 (RU_2_ and RL_2_). As a consequence of a strong precision grip, the bill and the tool become a rigid, combined structure, which also exposes the bill to a twisting, vertical bending and lateral bending moment with respect to its anatomical axes. Moments Mx and Mz should be far greater than any twisting moment My. Thus potentially dangerous levels of stress that might occur on the upper mandible (e.g. above the nares and at the nasal-frontal hinge, as indicated by the red oval) would be due to moments Mx and Mz. The NCC’s deep, short stout bill would be advantageous to counteract the forces and moments acting on it.

Thus the combination in the NCC of a deep, short, stout bill with a straight tomium on the upper mandible and an upwardly inclined tip on the lower mandible appears to be rare or absent in other species that forage in wood. Is the NCC’s bill morphology efficient for making and using tools? Probing with a tool places a unique combination of loading patterns on the bill to resist the forces and moments acting on the bill (Fig. 5). These forces and moments are highly likely to be in all directions at some stage during the manipulation of the tip of a tool in a probe site. According to Wolff’s law, or ‘bone functional adaptation’, bone tissue and overall bone structure adapt to changes in the mechanical loading environment^32^. Therefore, the NCC’s bill would be expected to have unique shape aspects and internal architecture that are a direct response to the unique loading patterns imposed by tool use (e.g. to deal with loading from all directions). Furthermore, the manipulation of raw material performed by NCCs in tool making may also place unique loading patterns on their bills. For example, snapping strong, fresh twigs in half to make hooked twig tools should place considerable torque on the bill^9^.

In a generalised *Corvus* bill for a given muscle force, a deeper upper mandible at its base will exert a greater bite force at a specific location along its length compared to the same location on a shallower upper mandible of the same length^22^. Likewise, bite force decreases from the base of the bill to the bill tip. Finch species specialised for seed cracking have very strong bite forces because of their deep, short and highly decurved bills^22^. NCCs are likely to require a considerable bite force to securely hold a tool during probing activity and raw material during tool manufacture. Their bill must counteract moments and forces associated with tool use that would almost always be greater than the force acting on the tool tip. This is because the required bite force becomes larger the further the tool tip is from the bill tip (Fig. 5). The NCC’s deeper upper mandible and shorter bill are both features that would act to increase bite force at the front of the bill. The deeper, stout structure of the caudal part of the NCC’s upper mandible (Fig. 1 & Fig. 4A) might also be advantageous for counteracting high (and possibly damaging) stresses due to forces and moments acting on a rigid bill to hold a tool securely during probing and for tool manufacture.

**Figure 6:**
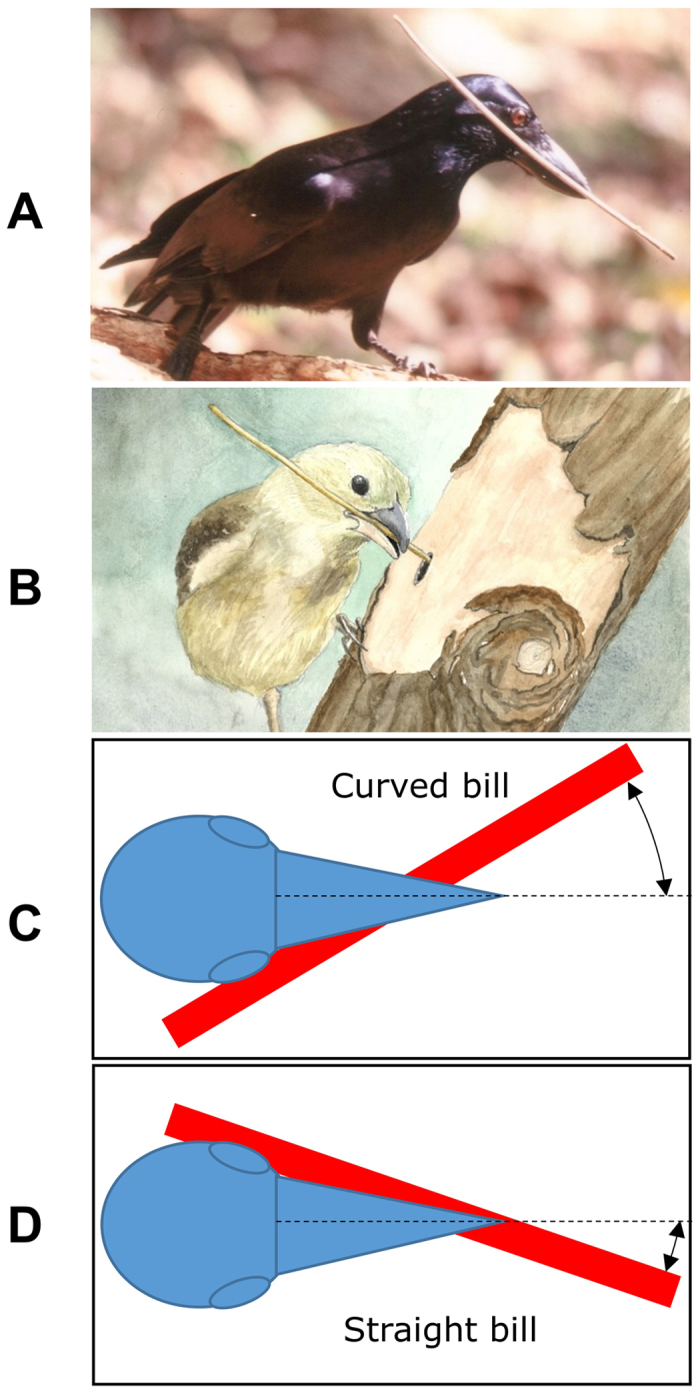
The advantage of a straight bill for holding a tool securely while placing the tool tip in the best possible position for its visually-directed manipulation. (**A**) A New Caledonian crow holding a stick tool angle-ways in its straight bill, which positions the tool tip as close as possible to the line of intersection between the mid-sagittal and mid-frontal planes of the bill. The tool is held securely by a strong bite from the tomia on the right side of the bill and a strong precision grip at the bill tip. (**B**) A watercolour based on a photograph of a woodpecker finch using a tool angle-ways in its decurved bill. A secure grip on a tool requires the woodpecker finch to position the tool more laterally in the bill, holding it with the tomia on both sides of the bill rather than the bill tip. This more secure holding method positions the tool tip further from the mid-sagittal plane of the bill, probably making fine manipulation of the tool tip more difficult. (**C**,**D**) Illustrations showing the distance of the tool tip from the mid-sagittal plane (dashed line) when a tool is held securely in either a decurved (**C**) or straight bill (**D**). The watercolour in B is reproduced with permission of Guido de Filippo.

Troscianko *et al.*^15^ suggested that the NCC’s straight bill is more efficient for visually-guided manipulation of a tool because it enables the tool tip to be positioned close to the mid-frontal plane of the bill. However, a more significant advantage of a straight bill is that it enables a tool to be securely held with the tip positioned in front of the bill as close as possible to the line of intersection between the mid-sagittal and the mid-frontal planes (Fig. 6A,D). To do this requires holding the tool angle-ways in the bill using only a relatively short rostral section of tomia on one side of the bill and the bill tip (Fig. 6A). Thus a strong bite would be highly advantageous to securely hold a tool in such a tenuous way to enable the best possible positioning of the tool tip for its efficient visually-directed control. At some sites NCC’s consistently make and use curved tools^8^,^33^,^34^. When they hold these tools angle-ways in their bills the curved end is usually the working end and orientated so that it curves in towards the mid-sagittal plane. Thus the working tip of a curved tool can potentially be placed in a better viewing position closer to the mid-sagittal plane than the tip of a straight tool.

Woodpecker finches use tools successfully with their curved bills by usually holding them pointing forward from the tips of their bills, not angled sideways^35^. This grip positions the tool tip close to the mid-sagittal plane but well below the mid-frontal plane due to the curvature of the bill, with the disadvantage of a poorer grip. To provide increased grip on the tool, a woodpecker finch must position the tool angle-ways in the bill and back from the bill tip (Fig. 6B). Although this positions the tool tip closer to the mid-frontal plane, it necessarily moves it sideways further away from the mid-sagittal plane (Fig. 6B,C). This would probably make fine manipulation of the tool tip more difficult due to requirements in visual/neuromuscular coordination. Also, the curved bills of large-billed crows trained to use tools prevented them from holding a tool close to the mid-sagittal plane^36^. Instead, they held the tool crossways with the tool tip positioned far from the mid-sagittal plane and used it in a sweeping action to retrieve food.

The tomia along the rhamphotheca of the NCC’s bill match closely the shape of the tomia of the underlying bone (compare Fig. 3A, B). As discussed above, the NCC’s straight upper tomium enables both a secure hold on the tool and the best possible positioning of the tool tip for its visually-directed fine manipulation. The relatively straight lower mandible further supports these two functional requirements, and provides a stronger grip on the tool because of the upwardly inclined tomium towards the front of the bill. The raised, strengthened tip of the lower mandible allows the bill to function similarly to the human opposable thumb and index finger to provide a two-jaw tip-to-tip precision grip^37^ (see Fig. 6A). The lower mandible may therefore be analogous to the robust human thumb, which is suggested to have been strengthened primarily to counter loading associated with tool use rather than tool manufacture^38^,^39^. Thus the NCC’s deeper, short bill and the dimorphically shaped tomia are consistent with the need to securely hold a tool in the front of the bill while it is used for probing, with the tool tip positioned in the best possible viewing position. A strong precision grip would also be advantageous for the fine manipulation involved in NCC’s tool manufacture. For example, for cutting across the strong fibres of *Pandanus* species leaves to make pandanus tools^12^ and the shaping of hooks on twig tools^9^.

Two additional structural features of the NCC’s bill are likely to counter the forces and moments acting on the bill associated with tool manufacture and use: the wider rami and the deeper and longer keel. The specific angle-ways holding of the tool that positions the tool tip away from the mid-sagittal plane means that forces acting on the working tip of the tool create a 3-dimensional moment around the point of support (i.e. lateral bending, vertical bending and twisting of the bill). The lateral widening of the rami is likely to provide better resistance to loading from different directions. The relatively long, deep and upturned keel demonstrates that the bill is not simply a scaled down generalised *Corvus* bill. The strengthened keel should help to withstand the increased stresses at the front of the bill where the bite force and precision grip are concentrated, thus avoiding micro-damage of the bone structure.

In summary, we provided both qualitative and quantitative evidence to show that the morphology of the NCCs’ bill is very different from the decurved bills of all other *Corvus* species, and possibly birds generally. The unique morphology cannot be explained by phylogeny. It is also extremely unlikely to have evolved from non-foraging-related morphogenesis to do with a founder affect or genetic drift; avian bills are generally under strong foraging-associated selection^22^ and there is no reason to suspect that the NCC’s bill is an exception. Furthermore, the NCC’s unique bill morphology is inconsistent with the bill morphology of other bird species that forage in wood with their bills or use tools. Our study indicates that the NCC with its unmatched tool-using lifestyle among birds has a bill much more specialised for tool manipulation. The morphological specialisation is multi-dimensional and makes the bill highly functional for tool manipulation compared to a decurved bill because of the ability to apply a strong precision grip while positioning the tool tip close to the line of intersection between the mid-frontal and mid-sagittal planes. Thus it is highly probable that at least some morphological aspects of the NCC’s novel bill evolved once the species began using tools, specifically for tool use. The implication being that a more specialised bill for tool use gave individuals greater foraging success when using tools and therefore increased fitness. We are not suggesting that the NCC’s bill shape is necessary for successful tool use as other bird species, such as the woodpecker finch, clearly accomplish this behaviour with very different bills. The tool-using woodpecker finch bill is decurved (Fig. 6B) and visually does not appear to be specialised for manipulating tools. Tool use in this species is mostly restricted to the dry season^40^, therefore a decurved bill may still be crucial for foraging when not using tools. In contrast, tool use in the NCC is practiced year-round. Detailed biomechanical analysis, which is beyond the scope of the current study, can be used to investigate whether the NCC’s bill is adaptive in some way. Specifically, whether the structure and shape of the NCC’s bill effectively counters the stress distribution acting on the bill when manufacturing and using tools.

A strong two-jaw thumb-to-finger precision grip in hominins is thought to have evolved for the efficient use of early simple tools rather than tool production^38^,^39^. Similarly, selection for enhanced visually-directed manipulation and a strong precision grip when using simple tools to extract prey was probably the main driving force for the NCC’s complex bill trait, which then enabled the fine manipulation required for their manufacture and use of complex hook tool designs^9^,^12^. This scenario would strengthen support for the idea that morphology adapted for enhanced tool manipulation is a prerequisite for subsequent increased sophistication of a species’ early tools^2^,^4^. Even the rapid evolution of different 3D bill shapes adapted to different foraging niches in Galápagos finches required interrelated changes in multiple genes over 2–3 million years^18^. Thus our findings are also the first morphology-based evidence that a nonhuman species may have evolved flexible tool behaviour over a potentially similar period of time.

## Methods

Our qualitative examination of the bill shape of all *Corvus* species (Supplementary Table S1, Fig. S1) confirmed that the NCC was exceptional in not having a decurved bill. Thus the main objective of the PCA was to identify the important shape characteristics of the NCC’s bill that set it apart from its close relatives. To do this, we could compare the NCC’s bill with the bills of a subset of *Corvus* species that provided a reasonable representation of the shapes of decurved bills in the genus. The nine species that we chose represented seven of the eight clades recently identified within *Corvus*^17^. Furthermore, our selection of the nine comparison species was conservative as three of them were in the same clade as the NCC, including *C. validus* which shared a recent common ancestor with the NCC (Supplementary Fig. S2). This would tend to reduce the chance of detecting important differences in the NCC. We analysed the bills of 36 specimens of 10 *Corvus* and also included one woodpecker species (*Dryocopus*) (Supplementary Table S2). The specimens of *Corvus*, which covered seven of the eight recognised phylogenetic clades (Supplementary Fig. S2), were five NCCs (*C. moneduloides*, Clade VII), five Daurian jackdaws (*C. dauuricus*, Clade I), five rooks (*C. frugilegus*, Clade IV), five carrion crows (*C. corone*, Clade VI), seven large-billed crows (*C. macrorhynchos*, Clade VIII), one grey crow (*C. tristis*, Clade VII), one long-billed crow (*C. validus*, Clade VII), one Australian raven (*C. coronoides*, Clade VII), one fan-tailed raven (*C. rhipidurus*, Clade V) and one white-necked crow (*C. leucognaphalus*, Clade II). The specimens of *Dryocopus* were four black woodpeckers (*D. martius*), comparable in body size to NCCs. The heads of the NCC and large-billed crow were fixed in 4% paraformaldehyde in 0.1 M Phosphate buffer solution, and the other specimens were preserved skins from the Yamashina Institute for Ornithology, Abiko, Japan.

We used a 3D landmark analysis to compare the overall bill shapes of the 11 species. Cross-sectional images of the head of each specimen were obtained with a micro-CT scanner (LaTheta LCT-100, Hitachi Aloka Medical, Tokyo, Japan). We next created a 3D reconstruction of each skull as a triangular mesh model based on the marching cube method, with an in-plane resolution of 0.2 mm/pixel and a gap size of 0.2 mm between images (image processing software Analyze 9.0, AnalyzeDirect, Overland Park, KS, USA).

**Figure 7:**
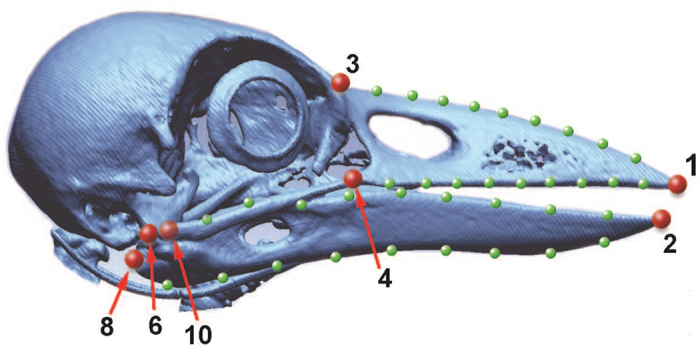
Landmarks and semi-landmarks on a 3D-reconstructed skull of a NCC. Large and small dots indicate landmarks and semi-landmarks, respectively. Only the landmarks in the mid-sagittal plane (1–3) and the right side of the bill (4, 6, 8 & 10) are shown. Landmarks are: 1, nasal-frontal hinge (*Foramen orbitonasale mediale*); 2, upper mandible tip; 3, lower mandible tip; 4 & 5, right/left rostral end of jugal bone (*Arcus jugalis*); 6 & 7, right/left caudal end of jugal bone (*Arcus jugalis*); 8 & 9, right/left caudal end of lower mandible (*Foramen pneumaticum*); 10 & 11, right/left articular fossa of the lower mandible (*Crista transversa fossae*, the unseen location is indicated by the transparent red dot).

Eleven primary landmarks of anatomically corresponding points were located on each 3D model of the bill using the software Geomagic XOS (3D Systems, Rock Hill, SC, USA) (Fig. 7). We located a total of 81 semi-landmarks evenly spaced along the curves between nine pairs of primary landmarks. The anatomical curves between landmarks were approximated by fifth-order Bézier functions to minimize the bending energy^41^ (Supplementary Document S1). Nine semi-landmarks were extracted along each approximated curve between the following pairs of landmarks; (i) the upper mandible tip and the nasal-frontal hinge, (ii) the upper mandible tip and the rostral end of jugal bone, (iii) the upper mandible tip and the caudal end of the jugal bone, (iv) the tip and the caudal end of the lower mandible, and (v) the tip and the *articular fossa* of the lower mandible (Fig. 7). Semi-landmarks along (ii)-(v) were extracted for both sides of the bill.

To standardize the size and orientation of the bills, represented by a set of 92 landmarks, we normalized the 3D datasets based on centroid size and superimposed them using a least-squares method (i.e., generalised Procrustes analysis) using the geometric-morphometry software Morphologika v. 2.5^42^. Equidistant landmarks are usually allowed to slide along curves or surfaces in geometric morphometrics since equidistance may not necessarily lead to geometrical correspondence of the landmarks across specimens^43^,^44^, resulting in possible artifacts in representing morphological variability. However, the position of the equidistant landmarks defined along the curves was found to move negligibly before and after sliding, thus having minimal affect on the extracted morphological variability^41^,^45^. Therefore, we did not slide the equidistant landmarks along the Bezier curves in the current study.

Principal Components Analysis (PCA) can be used to reduce a large set of variables to a smaller set that still contains most of the information in the larger set. To describe and quantify inter-species variation of the bills we carried out a PCA based on the variance-covariance matrix of the Procrustes residuals of all the bills^42^,^46^. To test the significance of the extracted Principal Components, we conducted an ANOVA test between species using statistical software R 3.0.3 (R Development Core Team); species with *n* = 1 were excluded from the analysis. To graphically show how each significant Principal Component contributes to overall bill shape, we created ‘high-value’ and ‘low-value’ wireframe models holding the values of all other Principal Components constant^41^.We quantitatively compared the curvature of the tomium (the inside edge) of the lower mandible among five *Corvus* species (carrion crow, Daurian jackdaw, large-billed crow, NCC and rook) and the black woodpecker. We calculated the profile of the bill curvature in the sagittal plane from the approximated Bezier curve fitted onto the tomium on each Computed Tomography slice along the length of the lower mandible. We used the radius length of the best-fit curve to indicate the degree of curvature (small and large radius lengths represent steep and shallow curves, respectively). Data were normalized using the length of the superior edge of the lower mandible (see Supplementary Document S1).

We used Computed Tomography imaging analysis to compare the NCC’s bill with the bills of two *Corvus* species that represented two general foraging methods within the genus. The rook represented a probing lifestyle and the large-billed crow an opportunist feeding lifestyle. The two NCC skulls (NCC 1 & NCC 2) were from wild-caught birds that had died unexpectedly in an outdoor aviary. The two rook skulls (Rook 1 & Rook 2) were obtained from a registered pest control operator after being culled in Hawke’s Bay, New Zealand. The two large-billed crow skulls (LaC 1 & LaC 2) were obtained in Tokyo following a culling operation. We scanned the six dried and degreased bills with the rhamphotheca removed.

The six skulls were scanned at the Auckland Radiology Group, Auckland, using a GE scanner (model Discovery CT750 HD) with an in-plane resolution of 0.14 mm/pixel and a gap size of 0.6 mm between images. The cranium and the upper mandible were scanned separately from the lower mandible. The Computed Tomography images of the upper mandible were separated from the cranium at the nasal-frontal hinge based on visual inspection. Total bone area for the upper and lower mandibles for each image was calculated using the plugin BoneJ in ImageJ. To compare the cross-sectional shapes of the rami of the lower mandibles, we calculated the minimum and maximum area moment of inertia (I_min_ & I_max_) using the image processing software ImageJ 1.48 v, together with the macro MomentMacroJ v1.4. I_min_ and I_max_ were measures of the minimum and maximum resistance to bending about the two principal axes of a 2D cross section. We then calculated the shape ratio I_min_/I_max_ to indicate the eccentricity of a 2D cross section, with I_min_/I_max_ = 1 corresponding to a circular shape that is equally resistant to bending in multiple directions^47^. We calculated I_min_/I_max_ values for the beam-like section of the right mandibular ramus from the rostral edge of the *Fenestra rostralis* to where the rami joined at the gonydeal angle (see Fig. 3).

To obtain an indication of the mechanical advantage of the three species’ bills, we also calculated the ratio of in-lever to out-lever length on the lower mandible^26^. In-lever length was measured from the *Crista intercotylaris* to the coronoid process, and out-lever length from the *Crista intercotylaris* to the tip of the mandible (measures taken on the right side of the mandible). A relatively longer in-lever distance compared to out-lever distance indicates a relatively shorter bill with a greater mechanical advantage thus enabling a stronger bite.

## Additional Information

**How to cite this article**: Matsui, H. *et al.* Adaptive bill morphology for enhanced tool manipulation in New Caledonian crows. *Sci. Rep.* **6**, 22776; doi: 10.1038/srep22776 (2016).

## Supplementary Material

Supplementary Information
