# Supplementary material for: Adaptive bill morphology for enhanced tool manipulation in New Caledonian crows
Source: Sci Rep. 2016 Mar 9;6:22776. doi: 10.1038/srep22776 (PMC4783770; doi:10.1038/srep22776)
Supplement: Supplementary Information [file srep22776-s1.pdf]

Adaptive bill morphology for enhanced tool manipulation in New Caledonian crows

Hiroshi Matsui, Gavin R. Hunt, Katja Oberhofer, Naomichi Ogihara, Kevin J. McGowan, Kumar Mithraratne, Takeshi Yamasaki, Russell D. Gray & Ei-Ichi Izawa

Supplementary Document S1

Calculation for approximating semi-landmark

The fifth-order Bezier curve was described as a polynomial function of parameter *u* and is given by:

$$\begin{aligned}\mathbf{p}(u) &= (1-u)^5\mathbf{p}_1 + 5u(1-u)^4\mathbf{p}_2 + 10u^2(1-u)^3\mathbf{p}_3 \\ &\quad + 10u^3(1-u)^2\mathbf{p}_4 + 5u^4(1-u)^1\mathbf{p}_5 + u^5\mathbf{p}_6 \\ &u \in [0,1]\end{aligned} \tag{1}$$

where $\mathbf{p}_i$, i = 1,..,6, were the position vectors of the control points that define the shape of the curve. Let $\mathbf{x}_k$, k = 1,..,N, be the digitized points along the curve. Approximation of the curve with the Bezier curve can be computed by solving the following least-squares minimization problem:

$$E = \sum_k (\mathbf{p}(u_k) - \mathbf{x}_k)^2 + \lambda \int (\frac{d^2\mathbf{p}}{du^2})^2 du \to \min \tag{2}$$

where E was the objective function, $\mathbf{p}(u_k)$ was the point on the curve closest to the kth digitized point $\mathbf{x}_k$ and λ was the weighting coefficient. The first term represented the fitting error between the points generated by the function and corresponding digitized points on the curve. The second term was the smoothness penalty function representing bending energy of the curve. By finding the values for the control points and u_k that minimized E, an accurate as well as smoothly fitted curve was obtained.

Calculation of the curvature of the superior edge of mandible

To compare the degree of the curvature of the superior edge of the mandible, we calculated the profile of curvature on the sagittal plane. Curvature normalized by the length of the superior edge can be calculated as:

$$c(u) = \frac{(p'_x p''_y - p'_y p''_x)L}{(p'^2_x + p'^2_y)^{1.5}}$$

where L is the length of the superior edge, and p'_x, p''_x, p'_y, and p''_y are dp_x/du, d^2p_x/du^2, dp_y/du, and d^2p_y/du^2, respectively (Hosaka, 1992). The length of the superior edge can be calculated as:

$$L = \int_0^1 \sqrt{(p'_x)^2 + (p'_y)^2 + (p'_z)^2}\, du$$

Reference

Hosaka M. (1992) Modeling of Curves and Surfaces in CAD/CAM. Springer, Berlin.

Supplementary Table S1

Table S1. Sources of images used to create the sketches of *Corvus* bill profiles in Supplementary Figure S1. The 43 *Corvus* species are those listed in the Handbook of the birds of the world (dos Anjos 2009, Reference 16 in the main text), excluding the New Caledonian crow.

Species	Source of image
C. albicollis	www.ibc.lynxeds.com
C. albus	www.ibc.lynxeds.com
C. bennetti	www.ibc.lynxeds.com
C. brachyrhynchos	www.ibc.lynxeds.com
C. capensis	www.ibc.lynxeds.com
C. caurinus	http://www.cykelkurt.com/fugle/eng/crows-of-the-world.html
C. corax	http://www.cykelkurt.com/fugle/eng/crows-of-the-world.html
C. cornix	www.ibc.lynxeds.com
C. corone	www.ibc.lynxeds.com
C. coronoides	www.ibc.lynxeds.com
C. crassirostris	www.ibc.lynxeds.com
C. cryptoleucus	www.ibc.lynxeds.com
C. dauuricus	www.ibc.lynxeds.com
C. edithae	www.ibc.lynxeds.com
C. enca	www.ibc.lynxeds.com
C. florensis	www.ibc.lynxeds.com
C. frugilegus	www.ibc.lynxeds.com
C. fusscicapillus	www.ibc.lynxeds.com
C. hawaiiensis	www.ibc.lynxeds.com
C. imparatus	www.sdakotabirds.com
C. insularis	http://bird-stockphotos.photoshelter.com
C. jamaicensis	http://www.cykelkurt.com/fugle/eng/crows-of-the-world.html
C. kubaryi	www.arkive.org
C. leucognaphalus	http://www.cykelkurt.com/fugle/eng/crows-of-the-world.html
C. macrorhynchos	http://www.cykelkurt.com/fugle/eng/crows-of-the-world.html
C. meeki	www.ibc.lynxeds.com
C. mellori	www.ibc.lynxeds.com
C. monedula	www.ibc.lynxeds.com
C. nasicus	www.alamy.com
C. orru	http://www.cykelkurt.com/fugle/eng/crows-of-the-world.html
C. ossifragus	www.ibc.lynxeds.com
C. palmarum	http://www.cykelkurt.com/fugle/eng/crows-of-the-world.html
C. pectoralis	www.ibc.lynxeds.com
C. rhipidurus	http://www.cykelkurt.com/fugle/eng/crows-of-the-world.html
C. ruficolis	http://www.cykelkurt.com/fugle/eng/crows-of-the-world.html
C. sinaloae	www.alamy.com
C. splendens	www.ibc.lynxeds.com
C. tasmanicus	http://www.cykelkurt.com/fugle/eng/crows-of-the-world.html
C. tristis	www.ibc.lynxeds.com
C. typicus	www.orientalbirdimages.org
C. unicolor	www.arkive.org
C. validus	www.ibc.lynxeds.com
C. woodfordi	www.ibc.lynxeds.com

Supplementary Table S2

Table S2. Specimens used for the PCA.

Species	English name	Specimen No.	Clade
Corvus moneduloides	New Caledonian crow	GT-4	Clade VII
		GT-8	
		4907	
		4911	
		4915	
C. dauuricus	Daurian jackdaw	YIO−55008	Clade I
		YIO−55009	
		YIO−55010	
		YIO−55013	
		YIO−55020	
C. frugilegus	Rook	2014−0201−1	Clade IV
		2014−0201−2	
		YIO−63513	
		YIO−55111	
		YIO−55116	
C. corone	Carrion crow	2012−1525	Clade VI
		2013−1220	
		2009−0881	
		2010−0357	
		2011−1049	
C. macrorhynchos	Large-billed crow	KO−1211A	Clade VIII
		KO−1211B	
		2012−1526	
		2013−0618	
		2014−0842	
		2014−0923	
		2014−0924	
C. leucognaphalus	White-necked crow	YIO−55142	Clade II
C. rhipidurus	Fan-tailed raven	YIO−55578	Clade V
C. tristis	Grey crow	YIO−55080	Clade VII
C. validus	Long-billed crow	YIO−55073	Clade VII
C. coronoides	Australian raven	YIO−55516	Clade VII
Dryocopus martius	Black woodpecker	YIO−23173	
		YIO−23176	
		YIO−23178	
		YIO−23179	

Supplementary Figure S1

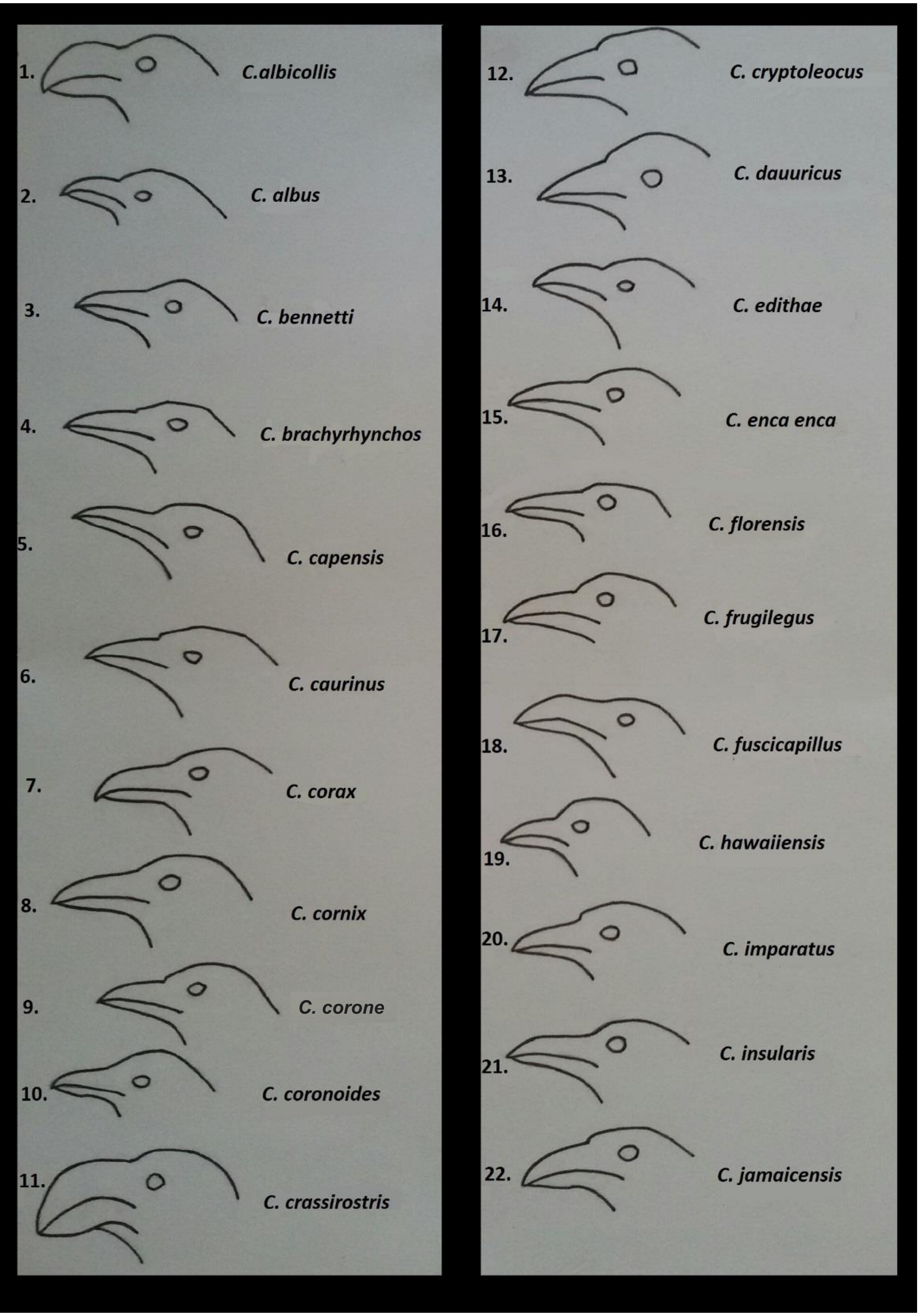

Supplementary Figure S1 (continued)

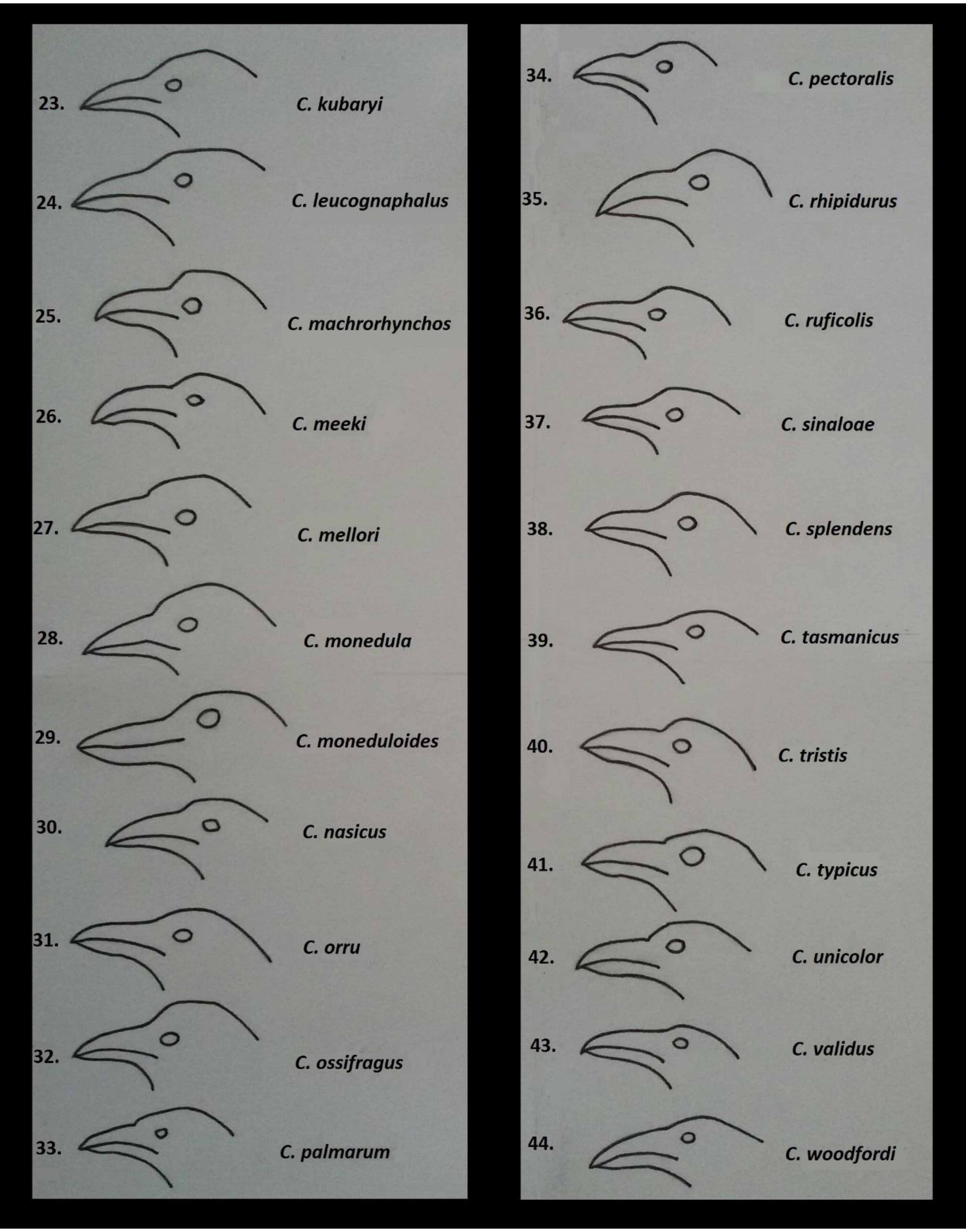

Figure S1. Profiles of *Corvus* species bills. The bill profiles are those of the 44 *Corvus* species described in the Handbook of the birds of the world (dos Anjos 2009, Reference 16 in the main text). All the profiles were drawn from photographs except for that of *C. meeki*, which was drawn from a drawing. The sketched profiles are not to scale or exact, but are provided to show that all species except the New Caledonian crow have decurved bills. Reproduced with permission of Guido de Filippo.

Supplementary Figure S2

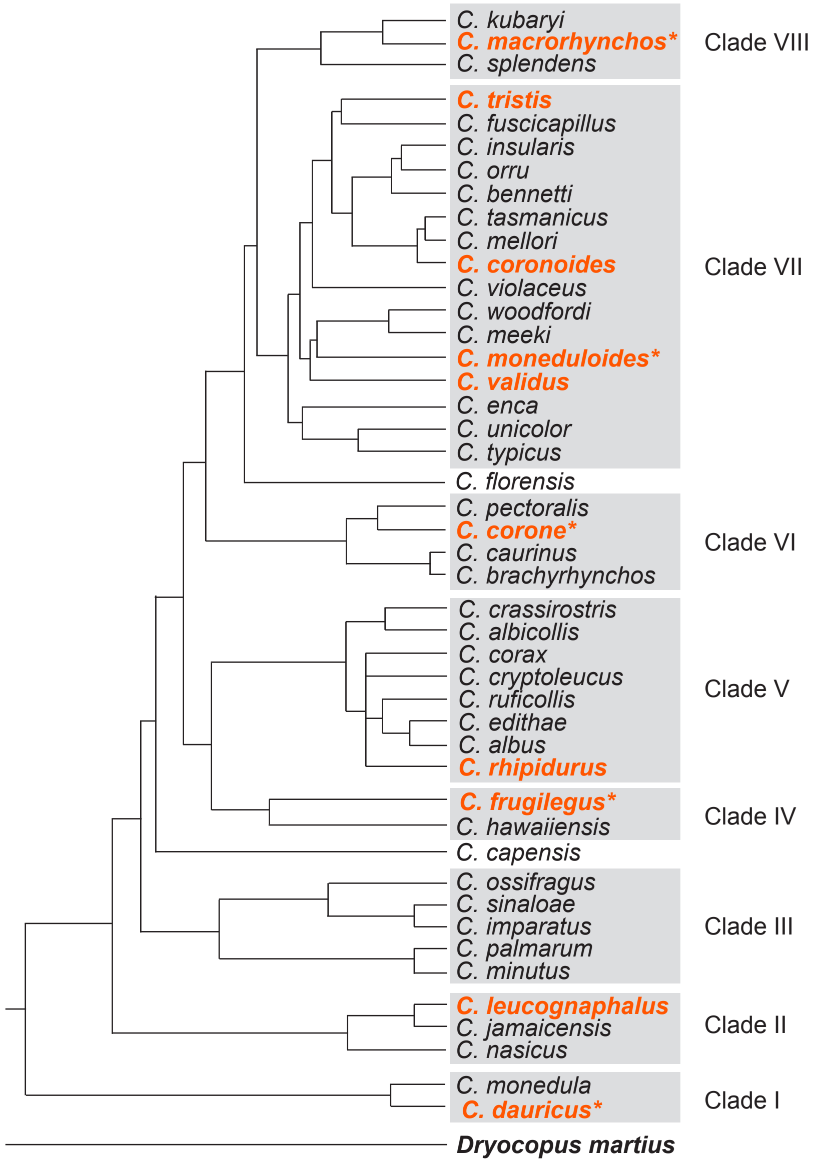

Figure S2. Phylogenetic relationships within the genus *Corvus* of the 10 species (in red lettering) used in the PCA. The phylogeny was taken from Fig. 2 in Jønsson et al. (2012, Reference 17 in the main text). An '*' indicates the species that were used to statistically compare the PC scores between species in the PCA and compare the curvature of the lower mandible.

Supplementary Figure S3

A

B

C

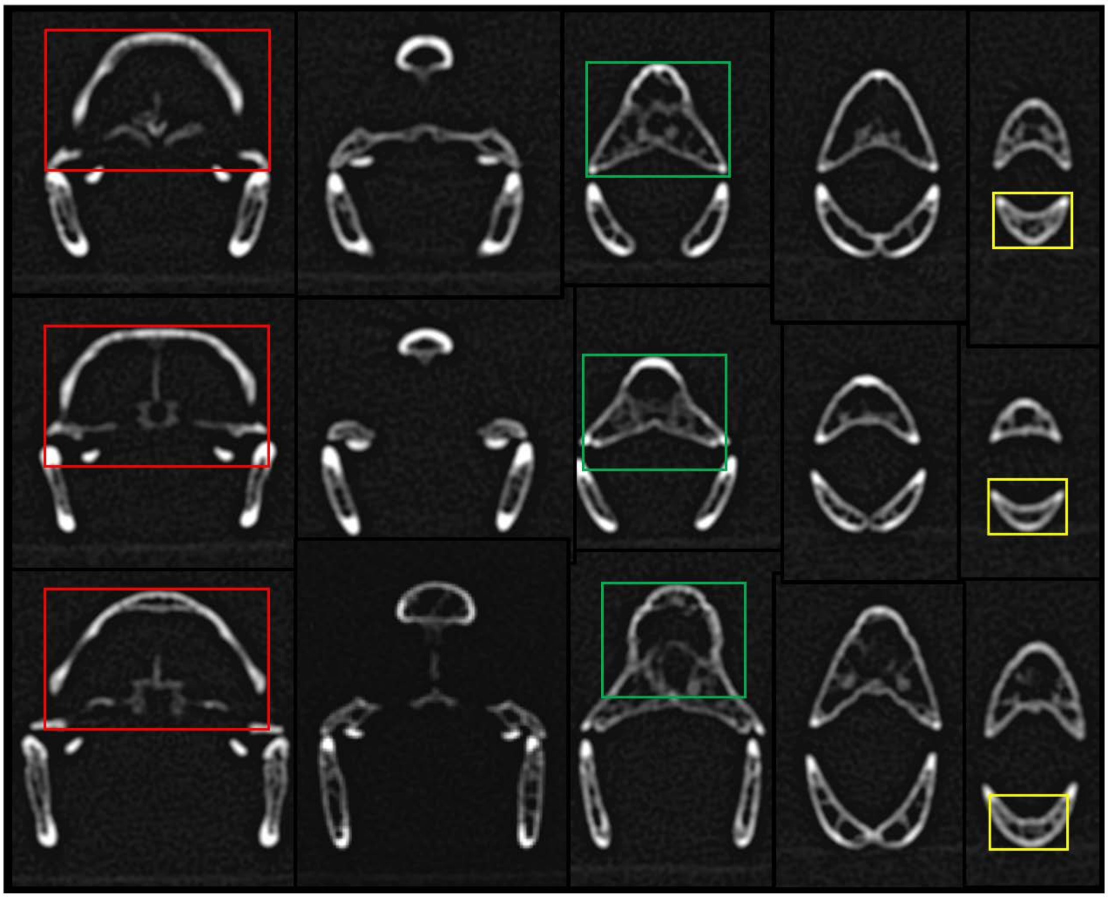

Figure S3. Selected CT cross-sectional images of the bills of three *Corvus* species. (A) A New Caledonian crow (NCC 2). (B) A rook (Rook 2). (C) A large-billed crow (LaC 1). From left to right the five cross sections for each species are (i) at the posterior edge of the nasal openings, (ii) midway between the posterior and anterior edges of the nasal openings, (iii) at the anterior edge of the nasal opening, (iv) at the gonydeal angle, and (v) midway between the gonydeal angle and the bill tip. The rectangles (the exact same size in each column) enable visual comparison of shape aspects between the three species. Note that the New Caledonian crow has a relatively deep upper mandible, deeper profile along the middle of the keel on the lower mandible and wider rami also on the lower mandible. These features provide a relatively stout bill with a squarer profile that should be better able to withstand forces in all directions. The whiter areas indicate regions of higher bone density.
